# Drivers of young adults’ voluntary compliance with COVID-19 protective measures: results from a multi-method study

**DOI:** 10.1186/s12889-022-14752-y

**Published:** 2022-12-21

**Authors:** Anne Reinhardt, Winja Weber, Constanze Rossmann

**Affiliations:** 1 Department of Communication, University of Vienna, Waehringer Str. 29, 1090 Vienna, Austria; 2 German Cancer Research Center, Im Neuenheimer Feld 280, 69120 Heidelberg, Germany; 3 Department of Media and Communication, Ludwig Maximilian University of Munich, Oettingenstr. 67, 80538 Munich, Germany

**Keywords:** Theory of planned behavior, COVID-19, Adolescents

## Abstract

**Background:**

With the easing of governmental COVID-19 restrictions, promoting voluntary public compliance with protective measures becomes essential for the pandemic evolution. A highly relevant target group for such health promotion are adolescents and young adults since they showed a strong decline in compliance throughout the pandemic. Building on an extended version of the Theory of Planned Behavior, this article investigates drivers of young people’s intentions to engage in voluntary COVID-19 measures in phases of re-opening.

**Methods:**

We conducted a sequential multi-method study among 14- to 29-year-olds in Germany: (1) a semi-standardized online survey (*N* = 88) to examine underlying beliefs and (2) a standardized online survey (*N* = 979) to identify influencing factors of compliance. The pre-study addressed the respondents’ perceptions about wearing a mask, social distancing, and avoiding crowded locations (open-ended questions). Responses for all protective measures were aggregated to identify general behavioral, normative, and control beliefs about COVID-19 protective measures. In order to gain generalizable insights into the factors determining voluntary compliance intentions in younger adults, we conceptualized the model constructs in the subsequent standardized online survey as formative measures based on their underlying beliefs. PLS-SEM was used to examine the effects of attitudes, subjective norms, perceived behavioral control, risk perceptions, and knowledge on young people’s intentions to comply (main study). Furthermore, a multi-group analysis was conducted to investigate differences between compliers and non-compliers.

**Results:**

The pre-study revealed that young people’s instrumental attitudes not only cover reasons of protection but also aspects of self-presentation (e.g., being a role model). The main study showed that besides knowledge and perceived severity of illness, instrumental attitude is the strongest predictor of intention to comply. The influence is even stronger in the group of non-compliers.

**Conclusion:**

This article highlights the importance of theory-based campaign planning and provides practical guidance to health communicators on how to increase voluntary compliance with COVID-19 protective measures in adolescents and young adults. The findings demonstrate the great potential of combining the Theory of Planned Behavior with risk perception and knowledge to gain deeper insights into the feelings and thoughts of younger target groups during a health crisis.

**Supplementary Information:**

The online version contains supplementary material available at 10.1186/s12889-022-14752-y.

During the last years, governments worldwide implemented several public health measures in order to stop the spread of the coronavirus. Due to an effective vaccination program and decreasing hospitality rates, these regulations are now drastically eased. However, at the same time, politicians and medical experts appeal to the people that voluntary adherence to protection behaviors (e.g., wearing a mask) remains important and might decide about the pandemic evolution in the future.

A subgroup of people who were found to show a strong decline in their compliance throughout the pandemic were adolescents and young adults [1]. Two reasons might explain their pandemic fatigue [2]: First, younger people have a lower risk of severe courses of COVID-19 than do older adults [3], resulting in lower risk perceptions [4, 5]. Secondly, behavioral recommendations such as social distancing counteract young people’s everyday lives, as they tend to have large social networks and an active social lifestyle [6–8] as well as a high need for independence [5]. Accordingly, younger people were more concerned about the negative effects of the pandemic on their social relationships and personal freedom than were older people [9].

To promote voluntary COVID-19 protection behaviors among adolescents and young adults, it is essential to gain deeper insights into this target group. Building on an extended version of the Theory of Planned Behavior (TPB) [10, 11], the present study aims at exploring the influence of instrumental and experiential attitudes, subjective norm, perceived behavioral control, risk perceptions, and knowledge on young people’s intentions to adhere to the measures in times of regulatory easing.

## A theory-based approach to health campaign planning

A critical component in developing an effective communication strategy is its theory- and evidence-based foundation. A well-examined theory by which to explain health behavior is TPB [12]. Accordingly, behavior is determined by intention, which, in turn, is guided by attitude toward the behavior, perceived subjective norm, and perceived behavioral control [13]. The attitude can be divided into two components [12]. While the experiential attitude describes one’s feelings toward a behavior (e.g., compliance with protective measures is stressful), the instrumental attitude refers to one’s cognitive evaluation of behavioral outcomes (e.g., compliance with protective measures is useful) [14]. Additionally, TPB suggests that attitudes, subjective norms, and perceived behavioral control are determined by specific beliefs [15]: behavioral beliefs (regarding positive/negative consequences of a behavior), normative beliefs (regarding expectations of important others), and control beliefs (regarding facilitating/inhibiting factors for performing a behavior). The more positive the attitude, subjective norm, and perceived behavioral control, the stronger the intention to engage in the behavior [12].

Over the past decades, numerous studies, including meta-analyses, addressed and confirmed the explanatory power of TPB ([11, 16, 17]; for recent meta-analyses in various health contexts, see [18–22]). Also, scholars have already successfully investigated preventive health behaviors through the lens of TPB in response to previous pandemics [23–25] and the coronavirus pandemic [13, 26–33]. It has been shown that compliance with social distancing was positively influenced by positive attitudes toward the behavior, a strong perceived behavioral control, and positive subjective norms [27, 33]. In addition, it has been shown that instrumental attitudes are a stronger predictor of the intention to comply than experiential attitudes [30, 34].

A recurring finding in these studies is that older people were more likely to adopt health precautions compared with younger people. This finding demonstrates the necessity to explore the age-specific cognitions of younger adults in more depth. However, research focusing exclusively on adolescents’ and young adults’ COVID-19 protection behaviors is scarce [13]. Moreover, none of the studies cited above examined the underlying behavioral, normative, and control beliefs in detail—even though they are essential to understanding the target group thoroughly and identifying effective messages promoting public compliance [16]. We ask:

RQ1: What are adolescents’ and younger adults’ behavioral, normative, and control beliefs regarding compliance with COVID-19 protective measures?

While TPB offers a valid basis for identifying levers of behavior change, it only covers the influence of beliefs, attitudes, perceived subjective norms, and perceived behavioral control. In contrast, risk perceptions, which are central in other theories of health behavior change such as Protection Motivation Theory (PMT) [35] or Health Belief Model (HBM) [36, 37], are only indirectly represented in the attitude, and only if individuals believe that a particular behavior is risky for themselves. However, it can be assumed that risk perceptions play a significant role during crises and should be observed explicitly [38]. Concerning the coronavirus outbreak, a few studies have already demonstrated that perceived health risk—for oneself or others—is an important determining factor for engagement in precautionary behaviors [39, 40]. In addition, studies found that it is worth investigating the influence of perceived susceptibility and severity of illness as two separate risk-related constructs: For instance, [31] showed that adherence to COVID-19 protective measures was affected by the TPB constructs as well as by the perceived vulnerability and perceived severity of illness, with the latter one in particular. Accordingly, the intention to comply with social distancing measures was influenced by perceived severity of COVID-19—but not by perceived susceptibility [41].

In addition to risk perceptions, knowledge was also found influence health-related behavior (as pronounced in the Integrated Behavioral Model; [16]). Experience from past health crises has already shown that awareness about and understanding of certain measures can facilitate adherence [42]. Similar evidence is also apparent in the context of the COVID-19 pandemic [43, 44]. For instance, [44] introduced knowledge as a predictor in an extended version of TPB. They found that knowledge of COVID-19 restrictions positively influenced intentions, mediated by positive attitudes. Therefore, while it can be expected that risk perceptions directly influence the intention to comply with protective measures, knowledge can be defined as a background factor indirectly influencing intentions through risk perceptions and attitudes.

**Fig. 1:**
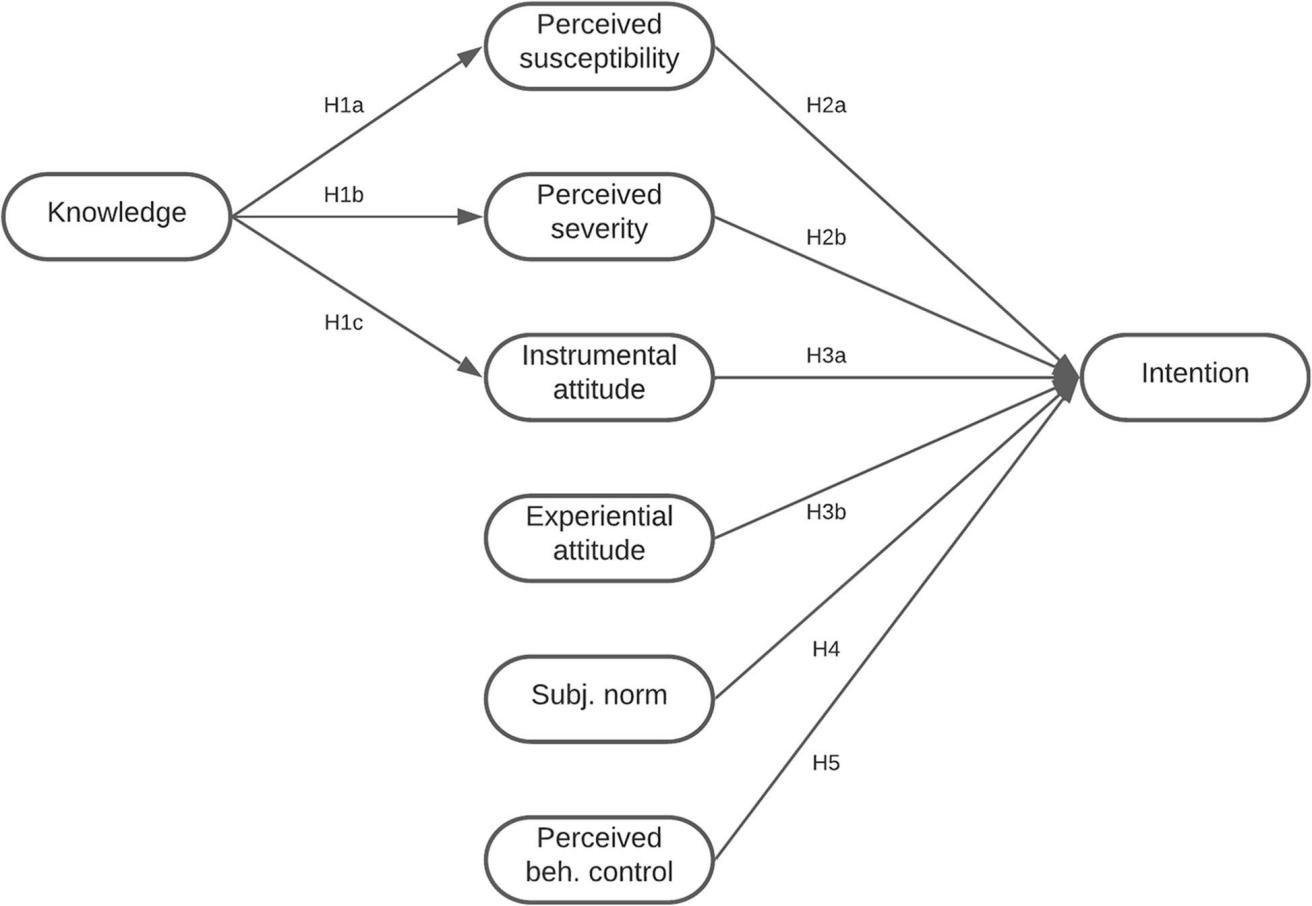
Theoretical model

Based on these considerations, we provide a model that combines TPB with risk-related constructs and knowledge (Fig. 1). In order to make claims about influencing factors of compliance after the lockdown phases, this study focuses on adolescents’ and young adults’ intentions to adhere to protective measures in times of regulatory easing. The following hypotheses are put forward:

H1: Knowledge has a positive, indirect effect on intention through (a) perceived susceptibility, (b) perceived severity, and (c) instrumental attitude.

H2: The (a) higher the perceived susceptibility and (b) the stronger the perceived severity, the stronger the intention to comply.

H3: The more positive the (a) instrumental and (b) experiential attitude, the stronger the intention to comply.

H4: The more positive the perceived subjective norm, the stronger the intention to comply.

H5: The greater the perceived behavioral control, the stronger the intention to comply.

To date, it is unclear whether the levers of COVID-19 compliance differ between those who show risky behaviors vs. those who adhere to the measures. For this reason, we will compare the hypothesized relationships between adolescents and young adults who showed high protection behavior in summer 2020 versus those who showed low levels of compliance. Summer 2020 represents a period with strongly loosened restrictions, making it an adequate indicator of behavior in the next pandemic phase with less stringent rules. We ask:

RQ2: How do the underlying processes differ between former compliers and non-compliers?

## Study design

A sequential multi-method study among people aged 14 to 29 was conducted to answer the hypotheses and research questions. The University of Erfurt Review Board approved all procedures. The first part involved a semi-standardized online survey to examine beliefs regarding COVID-19 protection measures in the target group (RQ1). Subsequently, the findings were used to inform a standardized online survey to identify influencing factors of intention (H1-H5, RQ2). We followed this multi-step approach since it can be expected that the observed phenomenon, i.e., voluntary compliance with COVID-19 measures, must be considered complex and “beyond the reach of a single method” ([45], p. 15). Moreover, it is in line with the TPB logic to examine the underlying beliefs in a first part, followed by a quantitative part focusing on model testing [46]. During both survey periods, German citizens were in the second lockdown phase (starting in November 2020). While schools and kindergartens mainly were opened, all other spheres of public life were drastically restricted (e.g., stay-at-home orders, contact restrictions).

## Part 1: semi-standardized online survey

### Method

#### Procedure

A semi-standardized online survey was conducted to answer the first research question. Participants were recruited in German schools and universities from the 1st to the 6th of December 2020 via snowball sampling. Participants provided online informed consent before completing the questionnaire. The open-ended questions addressed the respondents’ perceptions about wearing a mask, social distancing, and avoiding crowded locations.

#### Participants

A total of *N* = 88 completed the questionnaire (mean age M = 17.09, SD = 3.52; age range 14 to 28 years). A detailed description of participants can be found in Table 1.

**Table 1:** Characteristics of study sample (study 1)

	*N*	%
Gender		
Female	50	56.8
Male	36	40.9
X-gender	2	2.3
Educational status		
Low	59	67.0
High	33	33.0
Status		
Pupil (school)	56	63.6
Student (university)	11	12.5
Trainee	4	4.5
Employee	15	17.0
Unemployed	2	2.3

Note. *N* = 88

#### Measures

##### Behavioral beliefs

To identify behavioral beliefs regarding COVID-19 protective measures, participants had to indicate in an open text field which advantages and disadvantages of adhering to the protective measures they perceive (e.g., “What are the advantages of wearing a mask?”).

##### Normative beliefs

Relevant reference groups were assessed via the following questions: “Which people/groups of people think it is good if you adhere to the measures? What people/groups of people think it is not good?”

##### Control beliefs

To examine control beliefs, respondents indicated factors that would make it easier or more difficult for them to adhere to the recommended protective measures (e.g., “What kind of things or circumstances make it difficult or even impossible for you to wear a mask? And which factors would make it easier or help you doing it?”).

#### Analysis

Participants’ responses for all protective measures were aggregated to identify general behavioral, normative, and control beliefs about COVID-19 protective measures in the target group of young people.

### Results

Table 2 summarizes the results of the preliminary study. The behavioral beliefs covered both aspects of experiential and instrumental attitude. Regarding their experiential attitude, participants felt negatively affected mentally and physically. They felt “locked up at home” and suffered from the lack of contact with their friends. However, some of them also appreciated having more time for themselves (e.g., less stress). Regarding their instrumental attitude, participants stated that it is necessary to comply with the measures to protect themselves and others from infection and to contribute to an easier tracing of infection chains. In addition, some indicated reasons for self-presentation by stating that performing the behavior shows others that they take the pandemic seriously, which might encourage peers to follow suit.

**Table 2:** Results of the partially standardized preliminary study

Category	Subcategory	Considerations
Behavioral beliefs	Experiential attitude	- Negative impact on my mental health (e.g., concentration difficulties) - Negative impact on my physical health (e.g., lower fitness levels) - Lack of contact with my friends - I cannot do all the things in my free time that bring me joy - Feeling locked up at home - More time for myself/less stress
	Instrumental attitude	- Self-protection - Protection of others - To trace infection chains more easily - Self-presentation (To show others that I take the pandemic seriously)
Normative beliefs	Reference groups	- Parents - Friends - Life partners - Teachers/lecturers/bosses - Classmates/fellow students/colleagues - Older people (e.g., grandparents) - People from cultural/church/leisure groups (e.g., sports clubs) - Strangers I meet in my everyday life (e.g., salespeople) - Politicians/medical experts - Anti-COVID-19 protesters
Control beliefs	Facilitating factors	- Uniform regulations (e.g., between different federal states) - Stricter regulations - Frequent reminders (e.g., signs) - Expanding local transport (e.g., more buses than normally serve on a line) - Home-schooling/home office options - Virtual communication platforms and entertainment services (e.g., video chat, virtual concerts) - Free protective equipment (e.g., masks, disinfectant) - If others comply with the measures (e.g., keep their distance from me in the supermarket)
	Inhibiting factors	- To buy things for my everyday life (e.g., groceries) - My current housing situation

Relevant reference groups—both those who might find it good or bad if they adhere to COVID-19 protective measures—were parents, friends, and life partners. Other mentions concerned, inter alia, older people and experts (e.g., politicians).

Control beliefs were divided into facilitating and inhibiting factors for complying with COVID-19 protective measures. The first dimension includes uniform and strict governmental regulations as well as frequent reminders to comply with the measures. Additionally, participants found it facilitating working from home and using digital offers (e.g., FaceTiming) to get in touch with others. As an inhibiting factor, the current housing situation played a role (e.g., home office with kids).

### Discussion

The results underline the importance of openly assessing behavioral, normative, and control beliefs about COVID-19 measures since they are highly context-sensitive and target-group specific. The findings demonstrate that attitudes, perceived subjective norms, and perceived behavioral control are influenced by a great variety of formative beliefs [46]—which do not necessarily need to be positively correlated or correlated at all [47]. For instance, strict or uniform regulations might increase perceived behavioral control, while home-schooling options might not (e.g., because of the family situation). In the same way, a teenager might feel socially isolated because of the restrictions but simultaneously experiences less stress and no effects on a physical level. Lastly, the mentioned reference groups also covered both supporters and opponents of protection behaviors (e.g., even though vulnerable groups might find it good if adolescents and young adults adhere to the recommendations, parents or peers might not).

In order to gain generalizable insights into the factors determining voluntary compliance intentions with COVID-19 protection measures, we conceptualized the TPB constructs in the subsequent standardized online survey as formative measures based on their underlying beliefs instead of using the directly measured global TPB constructs. This decision was made for two reasons: First, a strength of this procedure is that it allows us to make precise claims about relevant aspects of compliance behavior, which is a crucial factor in identifying effective messages for health promotion. Second, based on the findings of our pre-study, relying on the directly measured variables brings problems in reliability. For instance, attitude is typically assessed in TPB studies using a semantic differential, including aspects such as “not helpful/helpful,” “bad/good,” and “wearing/not wearing.” However, based on the presented preliminary findings, it can be concluded that adolescents evaluate protective measures as a good idea for society but also as harmful to their personal life (e.g., concerning their desire for freedom). The same critique occurs for the global measurement of perceived behavioral control, which is usually assessed on an individual level (e.g., “It’s up to me, how easily I can comply with the preventive measures”). In our first study, though, we found that control beliefs predominantly covered aspects of governmental regulations (e.g., stricter roles, conform rules, free masks, etc.), which were not in the hands of the participants. Consequently, we decided to integrate the underlying beliefs in the model. However, the directly measured variables will be used to ensure the discriminant validity of the formatively measured constructs.

The study has several strengths, most notably its potential to gain deeper insights into adolescents’ and young adults’ thoughts and feelings about COVID-19 protective measures in Germany. Limitations include that the sample is not representative since we used snowball sampling and a relatively small sample. Thus, there is a high chance that some participants are part of the same social networks [48]. However, the procedure allowed us to efficiently reach adolescents and young adults from different educational backgrounds, which was highly relevant because younger people with lower educational status were found to show strong pandemic fatigue in particular [4]. Moreover, generalization of results was not the aim of this pre-study; instead, we aimed to get a broad picture of potential salient beliefs in this target group to inform the subsequent main study.

## Part 2: standardized online survey

### Method

#### Procedure

Participants were recruited between the 21st and the 30th of December 2020 via the online panel provider Gapfish. The study took place in Germany. The sample was stratified by education (50% lower/middle educational status vs. 50% higher educational status), gender (50% female vs. 50% male), and age (25% 14–16 years, 25% 17–20 years, 25% 21–24 years, 25% 25–29 years). This quota allocation ensured that our sample represented participants from different backgrounds equally. After giving informed consent, predictor and dependent variables were assessed.

#### Participants

A power analysis with g*Power ([49], version 3.1.9.6 for macOS) determined the sample size of *N* = 1000 (power: .95, f^2^ = 0.15). After data cleansing, the final sample size was *N* = 979. Participants were on average 21.10 years old (SD = 4.43) and 52.0% were female (*n* = 509). A full description of the participants may be found in Table 3.

**Table 3:** Characteristics of study sample (study 2)

	*N*	%
Gender		
Female	509	52.0
Male	470	48.0
Educational status		
Low	483	49.3
High	496	50.7
Status		
Pupil (school)	322	32.9
Student (university)	151	15.4
Trainee	123	12.6
Employee	313	32.0
Unemployed	62	6.3
Other	8	0.8
Urbanity		
< 20,000	405	43.3
≥ 20,000	510	56.7
Housing situation		
Alone	107	10.9
With family	561	57.3
With peers	255	26.1
Other	56	5.7

Note. *N* = 979

#### Measures

The following constructs were assessed (for an overview of descriptive analyses and measurement models, see Table 4):

**Table 4:** Overview of indicators and constructs

Construct	Measurement model	M (SD)
Intention (single item)	reflective	4.05 (0.81)
Instrumental attitude	formative	
Self-protection		3.98 (1.03)
Protection of others		4.18 (0.97)
Tracing of infection chains		3.69 (1.10)
To show others that I take the pandemic seriously		3.89 (1.03)
Experiential attitude	formative	
Negative impact on my mental health		2.49 (1.29)
Negative impact on my physical health		2.40 (1.26)
Lack of contact with my friends		2.68 (1.18)
I cannot do all the things that bring me joy		3.00 (1.24)
Feeling locked up at home		2.29 (1.31)
More time for myself/less stress		3.01 (1.20)
Subjective Norm	formative	
Parents		17.05 (6.84)
Friends		15.00 (6.88)
Life partners		14.34 (6.21)
Teachers/lecturers/bosses		14.92 (6.65)
Classmates/fellow students/colleagues		12.85 (6.14)
Older people (e.g., grandparents)		17.83 (6.99)
People from cultural/church/leisure groups		11.33 (5.97)
Strangers I meet in my everyday life		11.11 (6.22)
Politicians/medical experts		16.24 (7.10)
Anti-COVID-19 protesters		3.61 (4.37)
Perceived behavioural control	formative	
Uniform regulations		4.15 (0.96)
Stricter regulations		3.10 (1.19)
Frequent reminders		3.59 (0.88)
Expanding local transport		3.70 (1.09)
Home-schooling/home office options		3.92 (1.18)
Virtual communication platforms		3.86 (0.99)
Free protective equipment		4.08 (0.99)
If others comply with the measures		3.58 (1.21)
To buy things for my everyday life		1.94 (0.97)
My current housing situation		3.51 (1.01)
Perceived susceptibility (single item)	reflective	3.01 (1.08)
Perceived severity (single item)	reflective	2.60 (1.09)
Knowledge (score)	reflective	3.45 (3.71)

Note. *N* = 977–979

##### Intention

Participants’ intentions to adhere to the measures in phases of re-opening were measured by one question (“Please think about the time after the current lockdown: How often do you plan to adhere to the recommended protective measures?”, 1 = “never” to 5 = “always”) [10].

##### Instrumental attitude

Instrumental attitude was assessed based on the respective behavioral beliefs identified in the preliminary study (four items; 1 = “not at all” to 5 = “completely”).

##### Experiential attitude

Experiential attitude: Respondents were asked to indicate on a five-point Likert scale how strongly they agree with the respective statements (five items, 1 = “not at all” to 5 = “completely”).

##### Subjective norm

For each of the identified reference groups (Table 2), respondents had to indicate (a) how they believe these groups evaluate their compliance (1 = “very poor” to 5 = “very good”) and (b) to what extent they align their behavior with the opinion of the reference group (1 = “not at all” to 5 = “very strongly”). According to [10], both aspects were multiplied to form a score of subjective norm per reference group (1 = “very weak”, 25 = “very strong”).

##### Perceived behavioral control

The control-specific beliefs (Table 2) served as indicators by which to measure perceived behavioral control (10 items, 1 = “very inhibiting” to 5 = “very facilitating”).

##### Perceived susceptibility

Participants had to indicate their perceived susceptibility to getting infected with COVID-19 on a five-point Likert-scale (1 = “very unlikely” to 5 = “very likely”).

##### Perceived severity

In the same way, the perceived severity of COVID-19 for one’s health was assessed (1 = “not at all severe” to 5 = “very severe”).

##### Knowledge

Respondents’ knowledge of the coronavirus pandemic was measured via 11 items (inspired by [50]; for an overview of knowledge items, see Table 6). Participants indicated whether each statement is true or false/they do not know. Seven items contained accurate information, and four items presented noise. The knowledge score was calculated based on the sum of hits minus the sum of false answers (− 11 = “all answers wrong”, 11 = “all answers correct”).

##### Past behavior

For the comparison of compliers and non-compliers, participants indicated how strongly they adhered to the recommendations in summer 2020 (1 = “never” to 5 = “always”; eight items, including “wearing a mask”, “keeping distance”, “washing hands regularly”, “contact restrictions: friends”, “contact restrictions: family”, “contact restrictions: school/work”, “avoiding crowded public places”). The items were averaged to form a mean index of past behavior (α = .819, M = 3.64, SD = 0.74).

##### Global items

In order to run a redundancy analysis, all formatively measured constructs were additionally measured directly with a global item (1 = “do not agree at all” to 5 = “fully agree”) [51]. These are as follows: “It is good to follow the recommendations in order to stop the pandemic and its harms” (instrumental attitude), “It makes me feel sad and lonely to follow the recommendations” (experiential attitude), “People from my network expect me to comply with the protective measures” (subjective norm), and “If I want to, I can easily comply with the protective measures” (perceived behavioral control).

#### Analysis

To test our model (Fig. 1), we conducted partial least squares structural equation modelling (PLS-SEM, 10,000 bootstrap samples) using R (SEMinR package, version 2.3.0, [52]). The R script is openly accessible on OSF through https://osf.io/gjadn/?view_only=442b60898f8a4617903bc9dbff1ec5c9.

### Results

#### Model assessment

Before model evaluation, the reliability and validity of the constructs were evaluated [53]. All the formative indicators yield VIF values below the threshold of 3. To evaluate the relevance of the indicator weights, we followed the decision-making process for keeping or deleting formative indicators by [51] (weights should be significant or, at least, loadings should be significant; as a more conservative threshold, we deleted all indicators with loadings < |.30| to ensure that an indicator makes a sufficient contribution to forming the construct). As a result of this process, a few indicators had to be removed (experiential attitude, “Lack of contact with my friends”; norm: “Strangers I meet in my everyday life”; perceived behavioral control: “Expanding local transport”, “To buy things for my everyday life”, “Free protective equipment”, “If others comply with the measures”). Moreover, the redundancy analysis ensured convergent validity.

All other constructs were single-item measures. As we used well-established measures, it can be assumed that they are reliable and valid. For an overview of model assessment, see Supplement Tables S1 & S2.

#### Model of planned compliance with COVID-19 protective measures

**Fig. 2:**
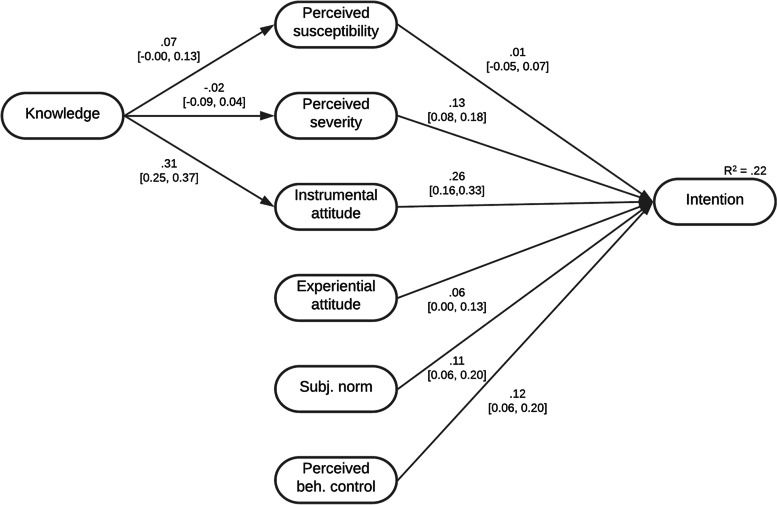
Path analytic model. Note: *N* = 979; standardized regression coefficients and 95% CI are displayed

The path model accounted for 21.9% of the variance in intention (R^2^_adj_ = .219, *p* < .001). Standardized parameter estimates are presented in Fig. 2. We found a small positive indirect effect of knowledge on intention through instrumental attitude (β = .08, T = 5.19, 95% CI: .05, .11), but not through perceived susceptibility and perceived severity (ns). The more knowledge participants possessed, the more positive their instrumental attitude and, in turn, the stronger their intention to comply. Hence, H1c was supported, while H1a and H1b were rejected.

Furthermore, we found support for H2b, indicating that adolescents and young adults who perceived a COVID-19 infection as more severe showed a stronger intention to adhere to the recommended protective measures (β = .13, T = 4.95, 95% CI: 0.08, 0.18). In contrast, perceived susceptibility had no effect on the intention to comply (ns). Therefore, H2a was rejected.

Instrumental attitude (β = .26, T = 5.75, 95% CI: 0.16, 0.33) and experiential attitude (β = .06, T = 1.75, 95% CI: 0.00, 0.13) were directly associated with intention. For experiential attitude, however, the regression coefficient is under .10, which is defined as a negligible effect size according to [54]. Thus, while H3a is fully supported, H3b can only be supported with reservations.

We further found a significant but small effect of subjective norm on intention (β = .11, T = 3.34, 95% CI: 0.06, 0.20), indicating that a positive subjective norm increases the intention to comply. Thus, H4 was supported.

Lastly, we predicted that a greater perceived behavioral control would lead to a stronger intention (H5). This hypothesis was confirmed (β = .12, T = 3.32, 95% CI: 0.06, 0.20).

#### Differences between compliers and non-compliers

In a second step, we conducted a multi-group analysis to examine differences between younger adults who showed lower and higher compliance levels in summer 2020. Participants were split into two groups based on their mean past behavior (non-compliers: M_past beh._ ≤ 3.50, *n* = 422; compliers: M_past beh._ > 3.50, *n* = 557). The multi-group analysis revealed similar patterns of effects as in the overall model (Table 5). However, the instrumental attitude is a significantly stronger predictor of intention in the group of non-compliers than in the compliers group (∆ β = .261, *p* < .01). In addition, the perceived susceptibility (∆ β = −.112, *p* < .05) as well as the perceived behavioral control (∆ β = −.148, *p* < .05) played a significant smaller role in people who did not comply vs those who complied. Notably, in the group of non-compliers, the effects were < |.10|; thus, they must be deemed as rather insignificant. All other paths did not differ on a significant level.

**Table 5:** Multi-group analyses (PLS-SEM)

Path	Non-compliers β	Compliers β	∆ β
Knowledge ➔ perceived susceptibility	.091	.035	.056
Knowledge ➔ perceived severity	−.049	−.038	−.011
Knowledge ➔ instrumental attitude	.291	.272	.019
Perceived susceptibility ➔ intention	−.045	.067	−.112*
Perceived severity ➔ intention	.150	.090	.060
Instrumental attitude ➔ intention	.360	.099	.261**
Experiential attitude ➔ intention	.042	.056	−.014
Subjective norm ➔ intention	.082	.182	−.100
Perceived beh. control ➔ intention	.042	.190	−.148*

Note. *N*_non-compliers_ = 422, *N*_compliers_ = 557, * *p* < .05, ** *p* < .01

#### Descriptive analysis of knowledge gaps

Since knowledge was found to be an important determinant of intention, we decided to take a deeper look into the knowledge items (Table 6). We found profound knowledge about vulnerable groups, preventive measures (e.g., hand washing, ventilation, social distance), and health risks. In contrast, only three-quarters of the sample knew how the coronavirus is transmitted. Interestingly, more than 70% of the sample overestimated the mortality rate by agreeing that it is at least 10%; the actual rate was around 2% in Germany at the end of 2020. Other knowledge gaps occurred regarding common COVID-19 myths: For instance, 40% of the sample agreed that infection with the coronavirus is just like getting the flu. Moreover, we found several believers of the widespread conspiracy theories that the virus was purposely created in Chinese laboratories (56.1% agreement) or that Ibuprofen increases the risk of getting infected (66.5% agreement).

**Table 6:** Descriptive analysis of knowledge items: correct knowledge

	%	*n*
Corona is not just like a flu (reversed)	60.3	590
Avoid using shared spaces at the same time as others	91.7	898
Virus was not created in Chinese laboratories (reversed)	43.9	430
Importance of proper ventilation	83.5	817
Symptoms are often only weak	57.0	558
Mortality is not at least 10% (reversed)	28.8	262
Severe complications may occur	84.7	829
Ibuprofen does not increase the risk of infection (reversed)	33.5	328
Transmission via mucosa	75.8	742
High relevance of hand washing	81.6	799
Older people are particularly affected	93.2	912

Note. *N* = 979

### Discussion

This study aimed to identify critical factors explaining adolescents’ and young adults’ voluntary adherence to COVID-19 protection measures in times of regulatory easing. Even though nowadays there are effective vaccinations against COVID-19, compliance with protective measures (e.g., wearing a mask) remains important. This becomes particularly evident when considering the current situation: While the Omicron variant spreads more quickly than earlier variants of the virus that cause COVID-19, an increasing number of breakthrough infections in vaccinated people occur [55]. However, in many countries globally, governments are easing the restrictions. For instance, since April 2022, wearing a mask is not mandatory anymore in the public in Germany; however, at the same time, German politicians strongly encourage citizens to adhere to protective measures voluntarily. Thus, there is a lot of leeway for personal decisions, which might determine the pandemic evolution in the following winter. It is therefore more important than ever to understand predictors of voluntary adherence to COVID-19 protection measures to create effective communication strategies addressing younger target groups.

We developed a theoretical model that combines TPB with risk perceptions and knowledge. After identifying the most salient beliefs among adolescents and young adults in a semi-structured pre-study, we conducted a large-scale national online survey among 14- to 29-year-olds. To our knowledge, this is the first TPB-based study in the context of the pandemic that not only tested the influence of relevant predictors on younger participants’ intentions to adhere to the protective measures but also assessed underlying beliefs. The findings demonstrate the great potential of this procedure.

We found that the willingness to comply with COVID-19 protection measures depends primarily on three factors: knowledge, perceived severity, and instrumental attitude. Specifically, (1) the more adolescents and young adults knew about the pandemic and respective protective measures, (2) the more severe they evaluated getting infected for their health, and (3) the more positively they evaluated the consequences of COVID-19 protective measures, the stronger their intention to behave in the recommended way—with knowledge indirectly affecting compliance through attitude. The findings are in line with previous research, demonstrating that instrumental attitudes [30, 34], risk perceptions [31], and knowledge [43, 44] are essential levers of COVID-19-related health behavior. Regarding risk perceptions, this article supports previous findings indicating that perceived severity is a significantly stronger predictor of compliance behaviour than perceived susceptibility (see also 41).

Knowledge gaps mainly concerned widespread misconceptions. For instance, nearly 40% of the sample assumed that COVID-19 is “as dangerous as the flu, no more and no less”— which is still one of the most mentioned anti-vaxxer-arguments in 2022— to be true. In addition, 56% believed that the virus was actively created in Chinese laboratories. The findings highlight the importance of debunking prominent false information and continuing to communicate facts about the pandemic.

People who believe in conspiracy theories and misinformation might not be reached with messages addressing knowledge anymore. However, not knowledge but the instrumental attitude was the strongest predictor of intention to comply, whereby the effect was even stronger in the group of non-compliers (for similar findings, see [30, 34]). Thus, messages promoting compliance with protective measures should strongly focus on positive adherence outcomes. Existing campaigns often address the instrumental attitude by highlighting aspects of protecting vulnerable groups. However, our pre-liminary study also found beliefs regarding self-protection and self-presentation salient in adolescents and young adults, and both beliefs turned out to be important indicators forming instrumental attitudes. Hence, health communicators could use these findings to keep voluntary compliance in young target groups high.

Furthermore, we found an effect of perceived behavioral control on intention. Because the relevant predictors were mostly related to policy aspects, this raises the question of whether perceived behavioral control will remain a relevant predictor in times of re-opening and regulatory easing. Future studies should investigate this relationship in more depth.

Subjective norm explained intention only to minimal extent. This was surprising because based on previous research (e.g., [27, 33]) we expected that adolescents and young adults rely strongly on the opinion of others—of their peers in particular. However, a meta-analysis on the explanatory power of TPB found that subjective norm is generally a rather weak predictor of intentions [56, 57], which was also confirmed in other studies in the context of the pandemic [13].

The findings of this study highlight the relevance of a theory- and evidence-based foundation when designing communication strategies for adolescents and young adults. According to our results, campaign messages addressing voluntary compliance with COVID-19 protective measures should focus on risk perceptions (e.g., using fear appeals or loss framing highlighting the implications of Long Covid). Moreover, we recommend emphasizing that young people can easily protect themselves by adhering to protective measures (e.g., using gain framing). Lastly, there should be more messages addressing the aspect of self-presentation, i.e., being a role model for others or showing friends and family members that one takes the pandemic seriously (e.g., using narratives, testimonials, or normative appeals). In addition, health information material should continue to debunk widespread misconceptions about COVID-19 (e.g., COVID-19 is just like the flu). To minimize the risk of boomerang effects such as psychological reactance, communicators could combine risk messages with factual information, positive messages, and cues to action to increase the perceived threat and simultaneously increase self-efficacy (e.g., using mixed message framing, [58]).

Findings of the main study should be interpreted in the light of several limitations. First, the analyses are based on cross-sectional data, which is why we only report intentions but not actual behavior. However, studies in the context of TPB found that intention is a strong predictor of behavior [59], which also proved to be true for compliance with COVID-19 protective measures [30].

Second, for methodological and practical reasons, we decided to use the underlying beliefs as formative indicators of attitudes, subjective norms, and perceived behavior control in our model—instead of the global, directly measured TPB constructs. To ensure that this decision was statistically correct, we used the directly measured variables in a redundancy analysis to evaluate the convergent validity of the formatively measured constructs. Since all paths became significant and showed T-values > 1.96, they can be deemed valid [53].

Third, the main study took place during the Christmas season, so there is a high chance that more people than usual did not adhere to the protective measures (e.g., social distancing) during this time. However, since the intention to comply with the protective measures in the time after the current lockdown—and not the actual behavior—served as a dependent variable, it can be assumed that the survey period did not affect our findings.

Fourth, the sample did not represent the German population aged 14 to 29 years because we decided to stratify the sample to ensure that different groups were equally represented.

Fifth, the model accounted for 22% of the variance in intention, which is relatively low (e.g., [22]). Compared to other TPB studies, we decided not to include past behavior as a direct predictor of intention in the model since it would not allow for new insights regarding levers of health behavior change (e.g., in terms of message design) [60]. Though, past behavior is indirectly displayed in the multi-group analysis of former compliers vs. non-compliers. By adding past behavior as a direct predictor to the model, we see a 5.4% increase in the explained variance (see Supplement Fig. S3).

The last limitation concerns the integration of the findings from study 1 in study 2: While both studies addressed adolescents and young adults with different educational statuses, the relative amount of school students was significantly higher in the preliminary study. However, the overall distributional pattern was similar in both studies, with school students being represented the most, followed by university students and employees. Therefore, we conclude that the assessed beliefs in the preliminary study adequately represent the beliefs of the sample in study 2.

## Conclusion

This article highlights the methodological and practical relevance of examining normative, subjective, and control beliefs before conducting TPB studies. By investigating the underlying beliefs, health communicators can more easily tailor their messages to promote voluntary compliance behavior in young audiences—which is still crucial given the easing of governmental restrictions by simultaneously very high infection rates. The findings demonstrate the benefits of using an extended version of the TPB as the theoretical foundation of health campaign planning. Hence, instrumental attitudes, risk perceptions, and knowledge are the most important levers of voluntary compliance, among non-compliers in particular. Regarding attitudes, positive messages are needed highlighting aspects of self-protection and self-presentation (e.g., being a role model). Moreover, a focus should be on educational campaigns to increase knowledge and enhance informed decision-making. Lastly, fear appeals, in combination with narratives, might be an appropriate tool to increase risk perceptions; however, we recommend to combine them with positive messages and cues to action to mitigate unintended boomerang effects.

## Supplementary Information

**Additional file 1: Table S1.** Test for indicator collinearity of formative constructs. **Table S2.** Test for indicator loadings of formative constructs. **Fig. S3.** SEM with past behavior as control variable.

## Data Availability

The datasets used and/or analyzed during the current study are available from the corresponding author on reasonable request.

## References

[1] COSMO (2021). Results of the COVID-19 Snapshot Monitoring COSMO: wave 04.

[2] WHO (2020). Pandemic fatigue reinvigorating the public to prevent COVID-19. Policy framework for supporting pandemic prevention and management. WHO Regional Office for Europe.

[3] CDC (2020). Severe outcomes among patients with coronavirus disease 2019 (COVID-19) - United States, February 12–march 16, 2020 [internet]. Centers for disease Control.

[4] Betsch C, Korn L, Felgendreff L, Eitze S, Schmid P, Sprengholz P (2020). COVID-19 Snapshot Monitoring (COSMO Germany) - Wave 24.

[5] Calmbach M, Flaig B, Edwards J, Möller-Slawinski H, Borchard I, Schleer C (2020). Wie ticken Jugendliche? Lebenswelten von Jugendlichen im Alter von 14 bis 17 Jahren in Deutschland [How do Youth in Germany tick?]. Bundeszentrale für Politische Bildung, editor.

[6] Albert M, Hurrelmann K, Quenzel G (2019). Jugend 2019–18. Shell Jugendstudie: Eine Generation meldet sich zu Wort [Shenn Youth Study] [Internet]. Beltz.

[7] Andrews JL, Foulkes L, Blakemore SJ (2020). Peer influence in adolescence: public-health implications for COVID-19. Trends Cogn Sci.

[8] Cohen AK, Hoyt LT, Dull B (2020). A descriptive study of COVID-19–related experiences and perspectives of a National Sample of college students in spring 2020. J Adolesc Health.

[9] German federal institute for risk assessment (2020). BfR-Corona-Monitor, 27.–28. Oktober 2020.

[10] Fishbein M, Ajzen I (2010). Predicting and Changing Behavior: The Reasoned Action Approach.

[11] Rossmann C, Bulck J (2020). Theories of reasoned action and planned behavior in media psychology. The international encyclopedia of media psychology [internet].

[12] Ajzen I (1991). The theory of planned behavior. Organ Behav Hum Decis Process.

[13] Godbersen H, Hofmann LA, Ruiz-Fernández S (2020). How people evaluate anti-Corona measures for their social spheres: attitude, subjective norm, and perceived behavioral control. Front Psychol.

[14] Wan C, Shen GQ, Choi S (2017). Experiential and instrumental attitudes: interaction effect of attitude and subjective norm on recycling intention. J Environ Psychol.

[15] Arafat Y, Mohamed Ibrahim MI (2018). The use of measurements and health behavioral models to improve medication adherence. Social and administrative aspects of pharmacy in low- and middle-income countries [internet].

[16] Montaño DE, Kasprzyk D, Glanz K, Rimer BK, Viswanath K (2015). Theory of reasoned action, theory of planned behavior, and the integrated behavioral model. Health behavior: theory, research, and practice.

[17] Rossmann C (2011). Theory of reasoned action. Theory of Planned Behavior. Baden-Baden: Nomos.

[18] Hagger MS, Chan DKC, Protogerou C, Chatzisarantis NLD (2016). Using meta-analytic path analysis to test theoretical predictions in health behavior: an illustration based on meta-analyses of the theory of planned behavior. Prev Med.

[19] Hagger MS, Polet J, Lintunen T (2018). The reasoned action approach applied to health behavior: role of past behavior and tests of some key moderators using meta-analytic structural equation modeling. Soc Sci Med.

[20] Li ASW, Figg G, Schüz B (2019). Socioeconomic status and the prediction of health promoting dietary Behaviours: a systematic review and Meta-analysis based on the theory of planned behaviour. Appl Psychol Health Well-Being.

[21] Riebl SK, Estabrooks PA, Dunsmore JC, Savla J, Frisard MI, Dietrich AM (2015). A systematic literature review and meta-analysis: the theory of planned Behavior’s application to understand and predict nutrition-related behaviors in youth. Eat Behav.

[22] Starfelt Sutton LC, White KM (2016). Predicting sun-protective intentions and behaviours using the theory of planned behaviour: a systematic review and meta-analysis. Psychol Health.

[23] Agarwal V (2014). A/H1N1 vaccine intentions in college students: an application of the theory of planned behavior. J Am Coll Heal.

[24] Yang ZJ (2015). Predicting young adults’ intentions to get the H1N1 vaccine: an integrated model. J Health Commun.

[25] Zhang X, Wang F, Zhu C, Wang Z (2019). Willingness to self-isolate when facing a pandemic risk: model, empirical test, and policy recommendations. Int J Environ Res Public Health.

[26] Adiyoso W, Wilopo. (2021). Social distancing intentions to reduce the spread of COVID-19: the extended theory of planned behavior. BMC Public Health.

[27] Gibson LP, Magnan RE, Kramer EB, Bryan AD (2021). Theory of Planned Behavior Analysis of Social Distancing During the COVID-19 Pandemic: Focusing on the Intention–Behavior Gap. Ann Behav Med.

[28] Hagger MS, Smith SR, Keech JJ, Moyers SA, Hamilton K (2020). Predicting social distancing intention and behavior during the COVID-19 pandemic: an integrated social cognition model. Ann Behav Med.

[29] Irfan M, Akhtar N, Ahmad M, Shahzad F, Elavarasan RM, Wu H (2021). Assessing public willingness to Wear face masks during the COVID-19 pandemic: fresh insights from the theory of planned behavior. Int J Environ Res Public Health.

[30] Norman P, Wilding S, Conner M (2020). Reasoned action approach and compliance with recommended behaviours to prevent the transmission of the SARS-CoV-2 virus in the UK. Br J Health Psychol.

[31] Prasetyo YT, Castillo AM, Salonga LJ, Sia JA, Seneta JA (2020). Factors affecting perceived effectiveness of COVID-19 prevention measures among Filipinos during enhanced community quarantine in Luzon, Philippines: integrating protection motivation theory and extended theory of planned behavior. Int J Infect Dis.

[32] Wollast R, Schmitz M, Bigot A, Luminet O (2021). The Theory of Planned Behavior during the COVID-19 pandemic: A comparison of health behaviors between Belgian and French residents. Delcea C. PLoS One.

[33] Yu Y, Lau JTF, Lau MMC (2021). Levels and factors of social and physical distancing based on the theory of planned behavior during the COVID-19 pandemic among Chinese adults. Transl Behav Med.

[34] Wang X (2022). Factors associated with public support for a lockdown measure in China during the COVID-19 pandemic. Asian. J Soc Psychol.

[35] Rogers RW. Cognitive and psychological processes in fear appeals and attitude change: a revised theory of protection motivation. Soc Psychophysiol Sourceb. 1983:153–76.

[36] Rosenstock IM (1974). The health belief model and preventive health behavior. Health Educ Monogr.

[37] Skinner CS, Tiro J, Champion VL, Glanz K, Rimer BK, Viswanath K (2015). The health belief model. Health behavior: theory, research, and practice.

[38] Brooke Rogers M, Pearce JM (2013). Risk communication, risk perception and behavior as foundations of effective National Security Practices. Strategic intelligence management.

[39] Joslyn S, Savelli S, Duarte HA, Burgeno J, Qin C, Han JH (2021). COVID-19: risk perception, risk communication, and behavioral intentions. J Exp Psychol Appl.

[40] Wise T, Zbozinek TD, Michelini G, Hagan CC, Mobbs D (2020). Changes in risk perception and self-reported protective behaviour during the first week of the COVID-19 pandemic in the United States. R Soc Open Sci.

[41] Callow MA, Callow DD, Smith C (2020). Older adults’ intention to socially isolate once COVID-19 stay-at-home orders are replaced with “safer-at-home” public health advisories: a survey of respondents in Maryland. J Appl Gerontol Off J South Gerontol Soc.

[42] Zhang L, Kong Y, Chang H (2015). Media use and health behavior in H1N1 flu crisis: the mediating role of perceived knowledge and fear. Atl J Commun.

[43] Al-Hasan A, Yim D, Khuntia J (2020). Citizens’ adherence to COVID-19 mitigation recommendations by the government: a 3-country comparative evaluation using web-based cross-sectional survey data. J Med Internet Res.

[44] Sturman D, Auton JC, Thacker J (2021). Knowledge of social distancing measures and adherence to restrictions during the COVID-19 pandemic. Health Promot J Austr.

[45] Morse JM, Niehaus L (2009). Mixed method design: principles and procedures.

[46] Ajzen I (2020). The theory of planned behavior: frequently asked questions. Hum Behav Emerg Technol.

[47] Chin WW (1998). Issues and opinion on structural equation modeling. MIS Q.

[48] Johnson TP (2014). Snowball sampling: introduction [internet].

[49] Faul F, Erdfelder E, Lang AG, Buchner A (2007). G*power 3: a flexible statistical power analysis program for the social, behavioral, and biomedical sciences. Behav Res Methods.

[50] Erceg N, Ružojčić M, Galic Z. Misbehaving in the Corona crisis: the role of anxiety and unfounded beliefs [internet]. PsyArXiv. 2020; Available from: https://osf.io/cgjw8. [cited 2020 Dec 28]. 10.1007/s12144-020-01040-4 PMC8280379 34305363

[51] Hair JF, Hult GTM, Ringle CM, Sarstedt M, Danks NP, Ray S (2021). Evaluation of formative measurement models. Partial least squares structural equation modeling (PLS-SEM) using R.

[52] Ray S, Danks N, Valdez AC (2022). SEMinR.

[53] Hair JF, Sarstedt M, Ringle CM, Gudergan SP (2018). Advanced issues in partial least squares structural equation modeling (PLS-SEM).

[54] Cohen J (1988). Statistical power analysis for the behavioral sciences.

[55] CDC (2022). Omicron Variant: What You Need to Know. COVID-19.

[56] Armitage CJ, Conner M (2001). Efficacy of the theory of planned behaviour: a meta-analytic review. Br J Soc Psychol.

[57] Manning M (2009). The effects of subjective norms on behaviour in the theory of planned behaviour: a meta-analysis. Br J Soc Psychol.

[58] Ort A, Reinhardt A, Koch L, Rossmann C (2021). The emotional effects of gain-loss frames in persuasive messages about sun protection on health promotional outcomes: evidence from an experimental study. Health Commun.

[59] Sheeran P (2002). Intention—behavior relations: a conceptual and empirical review. Eur Rev Soc Psychol.

[60] Sutton S (1998). Predicting and explaining intentions and behavior: how well are we doing?. J Appl Soc Psychol.

