# Supplementary material for: Drivers of young adults’ voluntary compliance with COVID-19 protective measures: results from a multi-method study
Source: BMC Public Health. 2022 Dec 21;22:2401. doi: 10.1186/s12889-022-14752-y (PMC9768790; doi:10.1186/s12889-022-14752-y)

Supplement

Table S1.

Test for indicator collinearity of formative constructs.

**Construct/item**	**VIF**
Instrumental attitude	
Self-protection	2.423
Protection of others	2.573
To trace infection chains more easily	1.604
To show others that I take the pandemic seriously	1.614
Experiential attitude	
More time for myself/less stress	1.128
Negative impact on my mental health (e.g., concentration difficulties) [reversed]	1.762
Negative impact on my physical health (e.g., lower fitness levels) [reversed]	1.590
Feeling locked up at home [reversed]	1.749
I cannot do all the things in my free time that bring me joy [reversed]	1.167
Subjective norm	
Parents	1.989
Friends	1.740
Life partners	2.254
Teachers/lecturers/bosses	2.088
Classmates/fellow students/colleagues	2.343
Older people (e.g., grandparents)	1.910
People from cultural/church/leisure groups (e.g., sports clubs)	1.973
Politicians/medical experts	1.689
Anti-COVID-19 protesters	1.082
Behavioral control	
Uniform regulations (e.g., between different federal states)	1.168
Stricter regulations	1.195
Frequent reminders (e.g., signs)	1.167
Home schooling/home office options	1.305
Virtual communication platforms and entertainment services	1.277
My current housing situation	1.097

Note. $N = 979$; VIF should be < 3.

Table S2.

Test for indicator loadings of formative constructs.

Path	b	T	95% CI
Self-protection → Instr. att.	0.883	28.124	0.811, 0.933
Protection of others → Instr. att.	0.807	19.500	0.716, 0.876
To trace infection chains more easily → Instr. att.	0.685	13.952	0.580, 0.773
To show others that I take the pandemic seriously → Instr. att.	0.853	23.946	0.773, 0.912
More time for myself/less stress → Exp. att.	0.823	10.268	0.615, 0.924
Negative impact on my mental health [reversed] → Exp. att.	0.552	4.793	0.287, 0.735
Negative impact on my physical health [reversed] → Exp. att.	0.616	5.716	0.367, 0.787
Feeling locked up at home [reversed] → Exp. att.	0.733	7.790	0.499, 0.867
I cannot do all the things that bring me joy → Exp. att.	0.411	3.169	0.118, 0.627
Parents → Norm	0.774	11.497	0.599, 0.862
Friends → Norm	0.585	7.440	0.401, 0.711
Life partners → Norm	0.463	7.230	0.395, 0.714
Teachers/lecturers/bosses → Norm	0.530	4.970	0.249, 0.614
Classmates/fellow students/colleagues → Norm	0.587	6.542	0.347, 0.662
Older people (e.g., grandparents) → Norm	0.591	7.214	0.394, 0.713
People from cultural/church/leisure groups → Norm	0.404	4.544	0.209, 0.556
Politicians/medical experts → Norm	0.831	14.162	0.672, 0.900
Anti-COVID-19 protesters → Norm	-0.352	-3.669	-0.523, -0.148
Uniform regulations → perceived beh. contr.	0.530	5.728	0.332, 0.693
Stricter regulations → perceived beh. contr.	0.865	17.207	0.733, 0.930
Frequent reminders → perceived beh. contr.	0.560	6.316	0.360, 0.709
Home schooling/home office options → perceived beh. contr.	0.382	3.947	0.174, 0.552
Virtual communication platforms → perceived beh. contr.	0.502	5.695	0.310, 0.651
My current housing situation → perceived beh. contr.	0.390	4.473	0.206, 0.548

Note. $N = 979$; indicator loading should be > |.50|; if not: at least, the loading should be > |.30| and significant.

Figure S3.

SEM with past behavior as control variable.

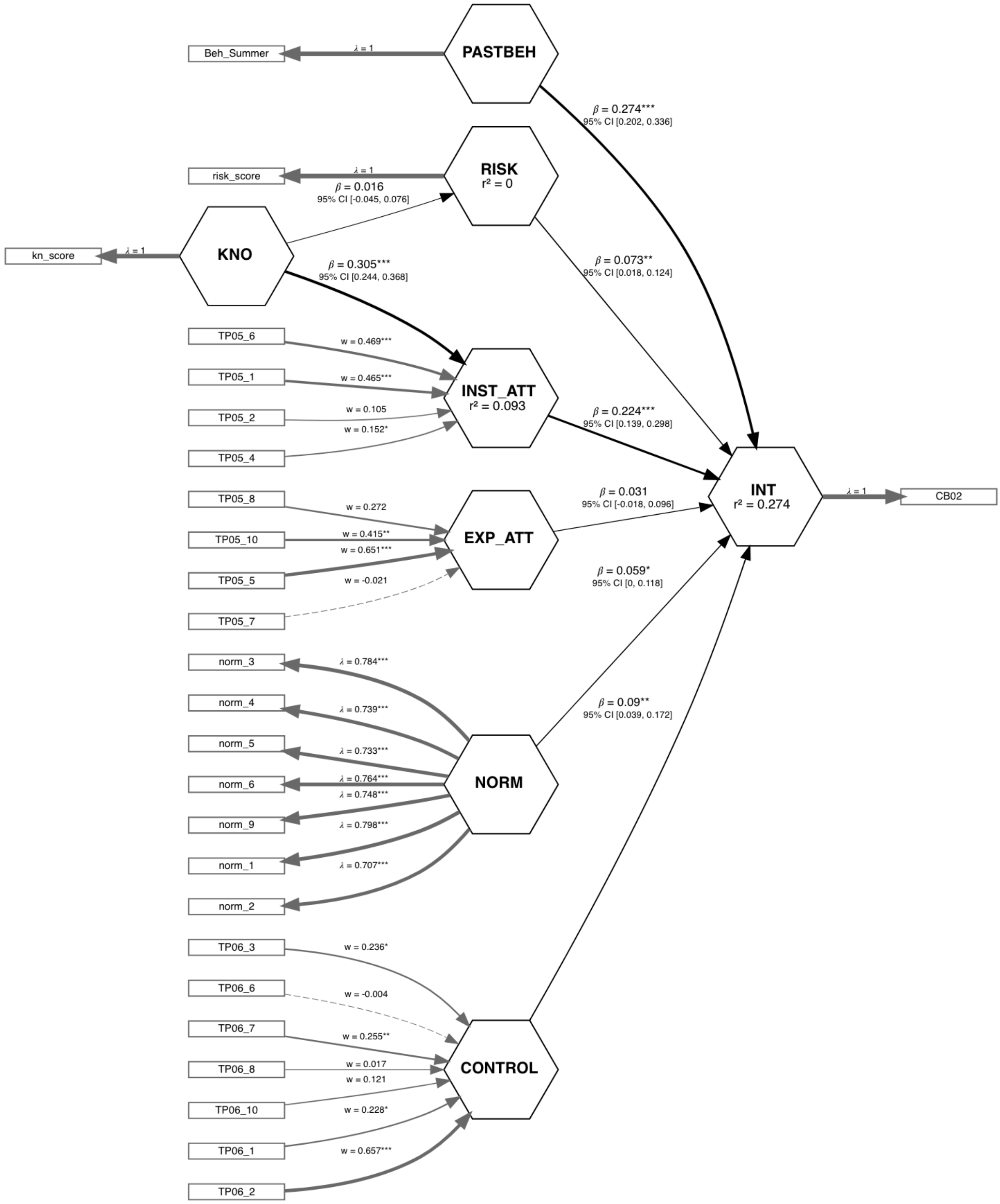

Supplement: Supplementary file 1 — Additional file 1: Table S1. Test for indicator collinearity of formative constructs. Table S2. Test for indicator loadings of formative constructs. Fig. S3. SEM with past behavior as control variable. [file 12889_2022_14752_MOESM1_ESM.pdf]
